# MicroRNA evolution, expression, and function during short germband development in *Tribolium castaneum*

**DOI:** 10.1101/gr.193367.115

**Published:** 2016-01

**Authors:** Maria Ninova, Matthew Ronshaugen, Sam Griffiths-Jones

**Affiliations:** Faculty of Life Sciences, University of Manchester, Manchester, M13 9PT, United Kingdom

## Abstract

MicroRNAs are well-established players in the development of multicellular animals. Most of our understanding of microRNA function in arthropod development comes from studies in *Drosophila*. Despite their advantages as model systems, the long germband embryogenesis of fruit flies is an evolutionary derived state restricted to several holometabolous insect lineages. MicroRNA evolution and expression across development in animals exhibiting the ancestral and more widespread short germband mode of embryogenesis has not been characterized. We sequenced small RNA libraries of oocytes and successive intervals covering the embryonic development of the short germband model organism, *Tribolium castaneum*. We analyzed the evolution and temporal expression of the microRNA complement and sequenced libraries of total RNA to investigate the relationships with microRNA target expression. We show microRNA maternal loading and sequence-specific 3′ end nontemplate oligoadenylation of maternally deposited microRNAs that is conserved between *Tribolium* and *Drosophila*. We further uncover large clusters encoding multiple paralogs from several *Tribolium*-specific microRNA families expressed during a narrow interval of time immediately after the activation of zygotic transcription. These novel microRNAs, together with several early expressed conserved microRNAs, target a significant number of maternally deposited transcripts. Comparison with *Drosophila* shows that microRNA-mediated maternal transcript targeting is a conserved process in insects, but the number and sequences of microRNAs involved have diverged. The expression of fast-evolving and species-specific microRNAs in the early blastoderm of *T. castaneum* is consistent with previous findings in *Drosophila* and shows that the unique permissiveness for microRNA innovation at this stage is a conserved phenomenon.

MicroRNAs are short nonprotein-coding RNAs, processed from hairpin precursors. MicroRNAs regulate gene expression by guiding the RNA-induced silencing complex (RISC) to complementary sites in the 3′ UTRs of target mRNAs, thereby inducing translational silencing and degradation (for review, see Bartel 2004). MicroRNA-target interaction usually requires base-pairing within a 6- to 7-mer “seed” region at the 5′ end of the mature microRNA, and thus each microRNA can potentially target hundreds of protein-coding genes (for review, see Bartel 2009). Consistent with their functional importance, seed sequences are the most highly conserved regions of the microRNA hairpins (Lai et al. 2003; Lim et al. 2003a,b). MicroRNAs were first identified for their role in the regulation of developmental timing in *Caenorhabditis elegans* (Lee et al. 1993; Reinhart et al. 2000) and were later shown to play important roles in various aspects of the development of both invertebrates and vertebrates (for review, see Kloosterman and Plasterk 2006). Most of our understanding of microRNA function in the development of arthropods comes from studies in the classical model organism *Drosophila melanogaster*, where they control essential developmental processes such as the clearance of maternally deposited transcripts, cell differentiation and apoptosis, morphogenesis, and organogenesis (Asgari 2013).

Despite its advantages as a model species, *Drosophila* development is not representative of the vast majority of arthropods. Fruit flies follow the so-called “long germband” developmental mode, which is derived and found only in a subset of holometabolous insect lineages (Peel 2008; Mito et al. 2010). The most common, and likely ancestral, mode of arthropod presegmentation development is short germband embryogenesis (Peel 2008; Mito et al. 2010). In short germband embryogenesis, a small number of cells in the blastoderm (called the germ anlage) form the most anterior embryonic segments, and the remaining portion gives rise to the extraembryonic serosal membrane (Handel et al. 2000). Whereas long germ development often occurs in a syncytium, more posterior segments in short-germband embryogenesis arise after gastrulation by growth and cell division, and patterning occurs in a cellularized environment via an oscillatory mechanism (Sarrazin et al. 2012). In many respects, short germband embryogenesis therefore more closely resembles the segmentation of vertebrate embryos.

The red flour beetle *Tribolium castaneum* is an emerging model organism that displays a number of ancestral features, including the short germband mode of development (Denell 2004; Richards et al. 2008; Roth and Hartenstein 2008). The availability of genetic tools and a wide range of embryonic patterning mutants have established *T. castaneum* as a model system to study this ancestral developmental mode (Denell 2004; Richards et al. 2008). *T. castaneum* has a fully sequenced genome (Richards et al. 2008) and an annotated protein-coding transcriptome (Kim et al. 2010). In addition, the morphology of its early embryogenesis is among the best characterized of the short germband insects (Handel et al. 2000, 2005; Denell 2004; Benton et al. 2013).

MicroRNAs are recognized as important players in developmental gene regulation, yet their expression and function in short-germband embryogenesis is poorly understood. Furthermore, little is known about the evolutionary constraints that act on microRNA developmental expression in general. We explored the microRNA complement of *T. castaneum* and expression of their targets in a developmental context, using a combination of small RNA and whole transcriptome RNA sequencing of successive time intervals covering beetle embryogenesis. We find that microRNA abundance markedly increases at the onset of zygotic transcription, both for conserved microRNAs and a large number of previously unannotated microRNAs organized in multiple rapidly evolving multicopy clusters. We show that maternally deposited protein-coding mRNAs that are down-regulated in the early embryo are significantly enriched in targets of these up-regulated microRNA families. We therefore show a role for early expressed microRNAs in maternal transcript clearance. Comparison with previous findings of microRNA-mediated maternal transcript degradation in *Drosophila* (Bushati et al. 2008) allows unprecedented insights into the evolution, expression, and function of microRNAs in the early stages of arthropod development.

## Results

### *T. castaneum* small RNA sequencing and annotation

*T. castaneum* embryonic development is significantly longer than that of fruit flies, spanning 6 d at 25°C. We collected embryonic samples from different time intervals after egg laying to determine the time points when key developmental events occurred (Supplemental Text; Supplemental Fig. 1). Based on these observations, we generated small RNA sequencing libraries from seven discrete intervals of *T. castaneum* embryogenesis covering the following key stages: very early embryo before the onset of zygotic transcription (0–5 h, herein referred to as “pre-ZT” embryos; note that zygotic transcription occurs at ∼8 h) (Supplemental Fig. 1B); later cleavage divisions and blastoderm formation (8–16 h); blastoderm differentiation and beginning of gastrulation (16–20 h); progressing serosal closure (20–24 h); elongating germband (24–34 h); fully segmented germband and appendage formation onset (34–48 h); and extended germband until hatching (48–144 h). We obtained between 3.5 and 6.5 million reads for each sample, over 85% of which mapped to the *T. castaneum* genome with no more than one mismatch.

**Figure 1.**
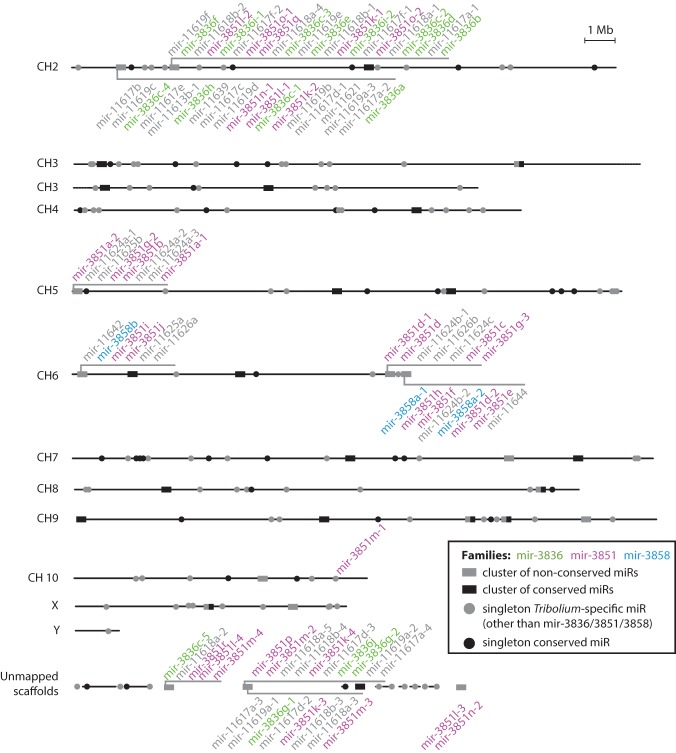
Genomic organization of known and novel microRNAs in *T. castaneum*. Horizontal lines represent the assembled chromosomes and the unmapped scaffolds of the *T. castaneum* genome assembly (r4.0), on which microRNA genes are found. MicroRNA-encoding loci are marked, and microRNAs within a 10-kb distance from each other are shown together as a cluster. Clusters encoding members of widespread *Tribolium*-specific microRNA families are labeled (see main text), and colors show paralogous family relationships.

The previously annotated set of microRNAs in *T. castaneum* comprises 203 hairpins that were experimentally identified in mixed adult and mixed embryonic data sets. Nearly half of these sequences have no identifiable orthologs in other species with sequenced genomes (Supplemental Table 1; Marco et al. 2010). We took advantage of the discrete embryonic stage data sets to search for putative novel microRNAs that may be expressed during narrow periods of time and thus escaped previous detection. We revised previous annotations and uncovered a total of 123 novel *Tribolium*-specific microRNA candidates using two independent approaches for novel microRNA identification (see Supplemental Table 1). A large fraction of these hairpins are paralogs of, or have similar extended seed sequences to, the microRNA families mir-3851 and mir-3836. Homology searches suggest that the mir-3851 family is *Tribolium*-specific (Supplemental Fig. 2). The miR-3836-3p has a seed sequence identical to that of the miR-3 family, but there is little similarity outside the seed, so homology cannot be confidently assigned. Regardless of their ancestral origins, the large number of paralogs from the mir-3836 family in *T. castaneum* suggests that they emerged by multiple lineage-specific duplications. Furthermore, there is a second set of novel sequences that lack homology with any known microRNAs. Many of these sequences are found in multiple copies and can be grouped in several families (Supplemental Fig. 2). Notably, the vast majority of mir-3851, mir-3836, and novel microRNA family members are organized in clusters in the genome, mostly localized in subtelomeric regions or unmapped scaffolds (Fig. 1). These clusters differ in their members’ copy number, sequence, and organization, suggesting rapid evolution by duplication and diversification. We collectively refer to these clusters as “multicopy microRNA clusters.”

### Small RNA temporal dynamics during *T. castaneum* embryogenesis

**Figure 2.**
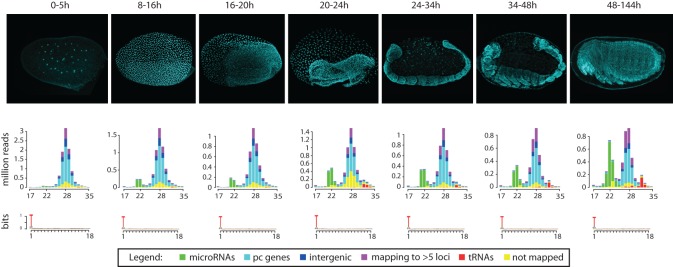
Small RNA types and size distributions throughout *T. castaneum* development. Histograms show the small RNA size and count distribution in each small RNA library (DAPI-stained representative images for the corresponding time intervals are shown in the *top* panels). Different colors reflect read mapping status: (yellow) not mapped, (purple) mapping to >5 loci, or (red) mapping to at least one tRNA, (green) microRNA, (cyan) protein coding (pc) gene, or (blue) intergenic regions. Sequence logos show the nucleotide bias for the first 18 positions of reads.

To gain insight into the developmental dynamics of small RNAs during *T. castaneum* development, we first assessed the overall content of the small RNA sequencing libraries of successive time intervals. Figure 2 shows the distributions of small RNA reads in each embryonic stage. Reads from all libraries and sizes display a very strong bias for uracil in the first position, which is typical for microRNAs and piRNAs. Size distributions of the sequenced reads display two peaks at ∼22 and ∼28 nt, with the vast majority of the ∼22-nt reads corresponding to known or newly annotated microRNAs. Thorough examination of the ∼28-nt fraction showed that these represent an abundant piRNA fraction (M Ninova, S Griffiths-Jones, M Ronshaugen, in prep.). The relative levels of putative microRNAs and piRNAs change markedly as development progresses. piRNAs are highly abundant in the earliest stages of development, consistent with their maternal deposition (Brennecke et al. 2008). MicroRNA levels, on the other hand, are initially very low but gradually increase and account for nearly 30% of all small RNA reads in the late embryo. These figures are in sharp contrast with the small RNA distributions observed in early embryonic developmental data sets from *Drosophila*, where microRNAs represent a significant fraction of the early embryonic small RNAs (see below) and dominate past the first couple of hours of development.

### Abundance, diversity, conservation, and 3′ end modification of maternally deposited microRNAs

**Figure 3.**
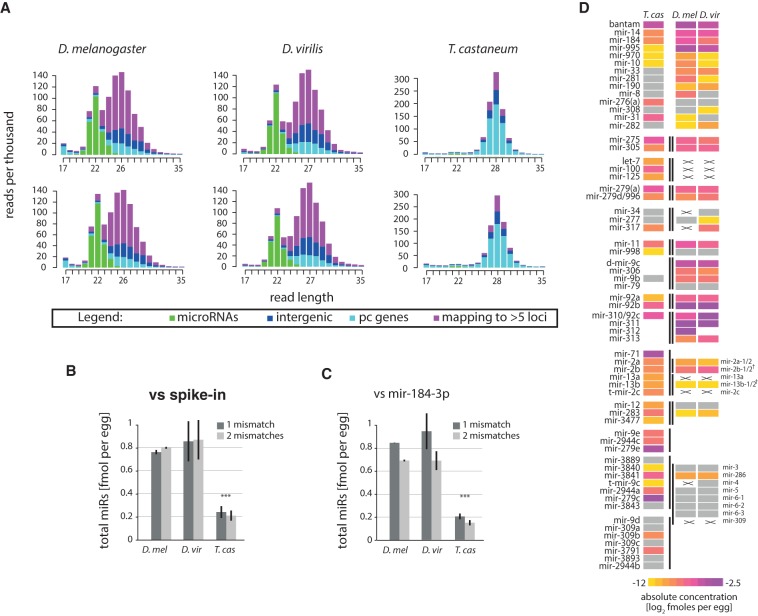
Quantities and conservation of maternally deposited microRNAs. (*A*) Read size and count distributions of small RNA sequencing libraries from oocytes in the three species (two biological replicates per species). Reads are colored depending on their mapping position to microRNAs (green), protein-coding (pc) (cyan), intergenic regions (blue), or multiple (>5) sites in the genome (purple). (*B*) Absolute abundance of total microRNAs as determined by deep-sequencing read counts relative to spike-in. (*C*) Absolute abundance of total microRNAs as determined by deep-sequencing read counts relative to miR-184-3p. In *B* and *C*, quantification from deep-sequencing analysis was performed using one and two allowed mismatches between read and genome. (*D*) Heat map showing the estimated concentrations of conserved microRNAs or clusters of microRNAs between Drosophilids and *T. castaneum*. MicroRNAs below an arbitrary threshold of 0.0002 fmol/cell are shown in gray. Lines mark clustered microRNAs. Crosses mark conserved microRNAs that are not expressed, while blank spaces denote absence of a given homolog from the corresponding cluster in a given species. MicroRNAs are aligned by homology; note that the *mir-3∼309* cluster of *Drosophila* has three homologs in *Tribolium*. (^†^) *mir-2* cluster in *Drosophila* has split.

We hypothesized that the apparent low microRNA levels in *T. castaneum* pre-ZT embryos reflects either insignificant maternal loading of microRNAs in oocytes or decreased sampling of microRNAs by sequencing due to high piRNA levels. We therefore sought to determine the absolute amount of microRNAs deposited in *Tribolium* and *Drosophila* eggs. To this end, we sequenced samples prepared from a fixed number of unfertilized eggs from *T. castaneum* and two divergent fruit fly species, *D. melanogaster* and *D. virilis*, with spiked-in synthetic 5′-phosphorylated oligonucleotides for normalization (see Supplemental Text; Methods). In addition, we quantified the absolute cellular levels of the abundant miR-184-3p and used it as an additional endogenous reference. miR-184-3p and spike-in levels were in a good agreement between qPCR and sequencing estimates, confirming that the sequencing data reflect well the microRNA abundances in this concentration range (Supplemental Text; Supplemental Fig. 3). Small RNA size distributions of the resulting libraries are shown in Figure 3A. As expected, *T. castaneum* oocytes show similar profiles to the transcriptionally inactive 0–5-h embryos, with highly abundant piRNAs. In contrast, *D. melanogaster* and *D. virilis* oocyte small RNA profiles display two prominent peaks corresponding to maternally provided microRNAs and piRNAs. MicroRNA read count normalization according to the endogenous and spiked-in references independently and consistently shows ∼0.2 fmol of microRNAs (∼120 million microRNA molecules) per egg in *Tribolium*, and approximately four times higher microRNA content in *Drosophila* (Fig. 3B,C). This difference in microRNA abundance between the fly and beetle is not sufficient to explain the difference in the microRNA/piRNA ratios in the two taxa (see Discussion).

We next assessed the most abundant maternally deposited microRNAs in *Tribolium* and compared these to fruit flies (Fig. 3D; Supplemental Table 2). The data show that the conserved microRNA families bantam, mir-275, mir-305, mir-14, mir-184, mir-995, mir-2/11/13, mir-92/310, mir-279, and mir-9 represent the most highly maternally loaded microRNAs in the insect oocytes. Clustered microRNAs are usually co-expressed, consistent with previous findings (Baskerville and Bartel 2005; Ruby et al. 2007; Ryazansky et al. 2011). A notable example of divergence of microRNA maternal deposition is the *mir-100/let-7/mir-125* cluster, members of which are loaded in *Tribolium* but not in *Drosophila*. In addition, the most abundant microRNAs in the beetle oocyte belong to two clusters*: mir-279e∼2944c* and *mir-3889∼3843*. These are diverged homologs of the *Drosophila mir-309∼6* and *mir-994∼318* (Ninova et al. 2014), whose maternal deposition in *Drosophila* is modest. The maternal microRNA complement of *T. castaneum* also contains a number of lineage-specific microRNAs, including the members of the massively duplicated 40 miRNA gene cluster on the X Chromosome, albeit at lower concentrations (Supplemental Table 2).

**Figure 4.**
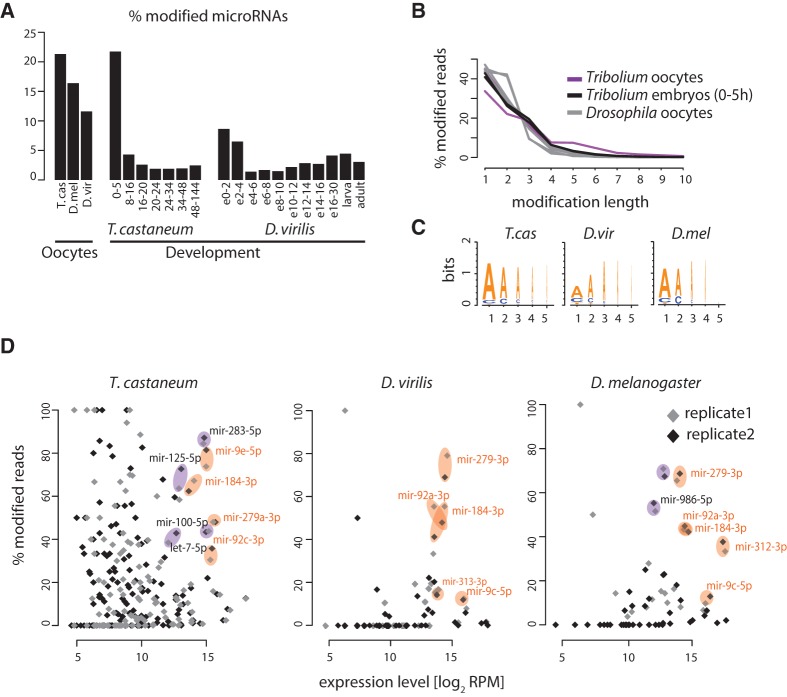
Nontemplated 3′-end nucleotides in oocyte microRNAs. (*A*) Proportion of microRNAs with nontemplate 3′-end nucleotide addition in oocytes and developmental small RNA libraries of *T. castaneum* and *Drosophila*. (*B*) Length distribution of nontemplate 3′-end nucleotide additions. (*C*) Sequence logos showing the most frequent nucleotides in the first five positions of the 3′-end nontemplated nucleotide additions in the three species. (*D*) Scatter plots showing the proportion of reads with nontemplate 3′-end additions as a function of the total read counts (modified and nonmodified) per million for each microRNA. Results of different replicates for each species are shown separately. Example singleton microRNAs or microRNAs from clusters that are conservatively deposited in the egg are highlighted in orange, and highly deposited and strongly modified microRNAs specific for a given species are highlighted in purple.

MicroRNA 3′ ends are known to be subject to various modifications in different model systems, including ligation of additional nucleotides to the 3′ end of the mature product (Ha and Kim 2014). A recent study reported that up to 30% of microRNAs in the oocytes and early embryos of *D. melanogaster* and deuterostomes are 3′ end-modified, and in *D. melanogaster* this modification was suggested to be involved in maternal microRNA clearance (Lee et al. 2014). We addressed the presence and developmental dynamics of microRNA 3′-end nucleotide additions in *T. castaneum* deep-sequencing data, together with a *D. virilis* developmental time series previously generated by our group for comparison (Ninova et al. 2014). Because we can only detect 3′ end addition of nucleotides different from the ones at the genomic position immediately after the microRNA 3′ end cleavage site, our estimates of the extent of microRNA modifications in insect oocytes are likely to be conservative. Results showed at least 20% of the maternally deposited microRNAs in *T. castaneum* are 3′ end-modified (Fig. 4A), which is significantly higher than later times or in other tissues, consistent with the previous observations in *D. melanogaster* (Lee et al. 2014). The majority of nontemplate 3′ end additions are between 1 and 3 nt in length (Fig. 4B) and are almost exclusively adenosines (Fig. 4C). The proportion of reads with modified ends varies between different microRNAs and is poorly correlated with expression level—while some highly abundant microRNAs are not significantly modified, several display a particularly high ratio between their modified and unmodified forms in oocytes (Fig. 4D). These observations are again consistent with those in *D. melanogaster* (Lee et al. 2014). Notably, among the conserved maternally loaded microRNAs in *Drosophila* and *Tribolium*, products of the same families (e.g., miR-184-3p, miR-279-3p, miR-9-3p, miR-92/310-313-3p) are heavily modified. Thus, the process of oligoadenylation of specific maternally deposited microRNAs is conserved between the two insect taxa.

### Developmental expression of conserved and nonconserved microRNAs

**Figure 5.**
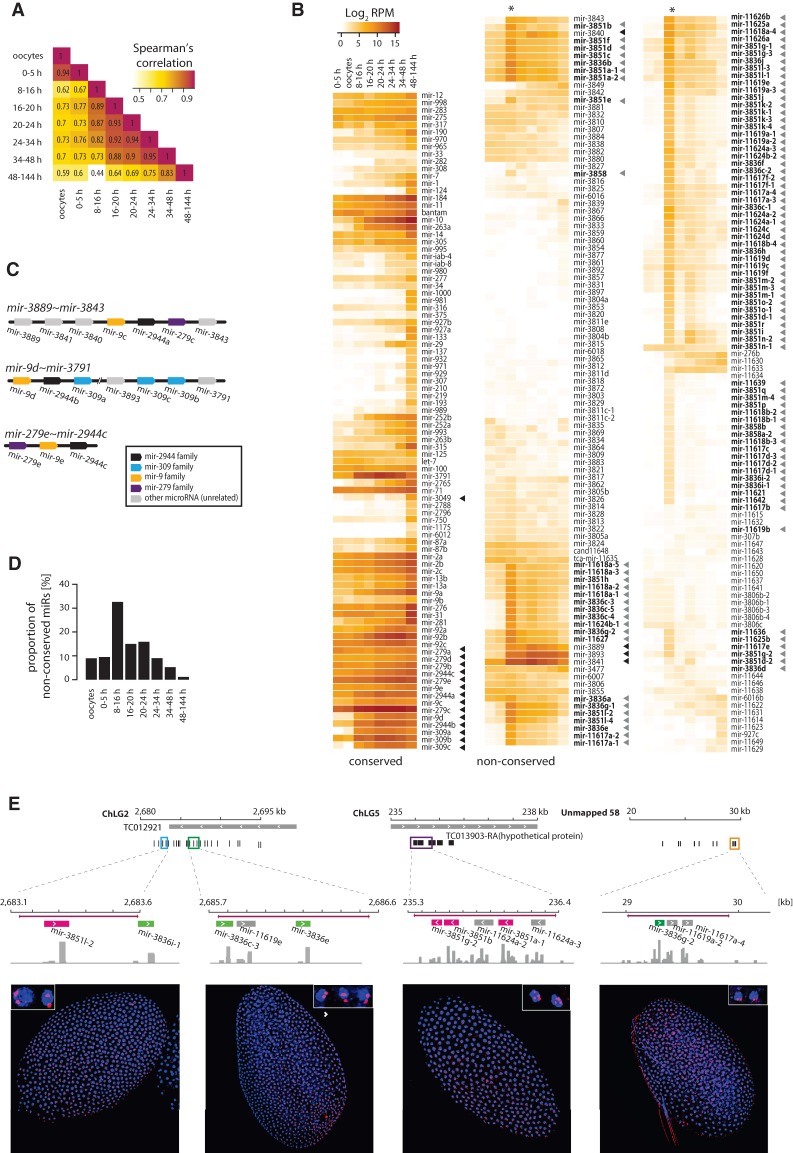
Developmental expression of conserved and nonconserved microRNAs throughout *T. castaneum* development. (*A*) Heat maps representing Spearman's correlation values for all-versus-all comparisons of the microRNA expression levels in oocytes and embryonic intervals of 0–5, 8–16, 16–20, 20–24, 24–34, 34–48, and 48–144 h. (*B*) Heat maps showing the normalized expression levels (reads per million) of conserved and species-specific microRNAs in *T. castaneum* oocytes and developmental intervals. Gray arrowheads indicate microRNAs from the mir-3851 and mir-3836 families and other microRNAs clustered in the same loci, as in Figure 1. Black arrowheads mark members of the *mir-279e∼2944c*, *mir-3889∼3843*, and *mir-9d∼3791* clusters. (*C*) Diagram of the genomic organization of the *T. castaneum mir-279e∼2944c*, *mir-3889∼mir-3843*, and *mir-9d∼3791* clusters. MicroRNAs are color-coded based on sequence homology. (*D*) Relative proportion of *Tribolium*-specific microRNA reads at different developmental stages. (*E*) Spatial expression of *Tribolium-*specific microRNA clusters detected by in situ hybridization. *Top* diagrams indicate the genomic regions encoding *Tribolium*-specific microRNAs used for antisense DIG-labeled RNA probe design, with probe regions in colored boxes. MicroRNA genes are black or color-coded based on homology to *mir-3851* (magenta) and *mir-3836* (green). Histograms represent coverage tracks generated with IGV. (*Bottom*) Confocal images of *T. castaneum* blastoderm embryos showing nascent microRNA transcripts (red); blue represents DAPI nuclear staining.

We next investigated the temporal dynamics of microRNA expression throughout beetle development. MicroRNA read counts from the different small RNA sequencing data sets are shown in Supplemental Table 2. Figure 5A shows the correlations of microRNA expression profiles between the different developmental stages of *Tribolium* in an all-versus-all manner. As expected, the microRNA repertoire in oocytes and 0–5-h embryos is highly similar, as zygotic expression is not active during the initial cleavage divisions. The similarity between pre-ZT and blastoderm embryos (8–16 h), however, is significantly lower, indicating a shift in the microRNA expression profile upon activation of zygotic transcription. Subsequently, the correlation of microRNA expression is the highest between neighboring stages, and similarity decreases with increasing developmental distance. Thus, developmental transitions in *T. castaneum* are accompanied by shifts in the global microRNA profiles.

To gain further insight into the diversity and dynamics of the microRNA complement throughout beetle embryogenesis, we assessed the normalized expression levels of the annotated *T. castaneum* microRNAs at each developmental interval. Heat maps in Figure 5B show the levels of individual microRNAs grouped by conservation in other species. Consistent with previous notions, conserved microRNAs are generally more highly expressed (Ruby et al. 2007; Liang and Li 2009; Roux et al. 2012; Meunier et al. 2013). We detect virtually all conserved microRNAs during at least one stage of development, with the vast majority displaying their highest levels in the late embryo during morphogenesis and organogenesis (48 h–6 d). The most strongly expressed microRNAs during the early and intermediate stages of embryogenesis derive from three clusters, *mir-9d∼3791*, *mir-3889∼3843*, and *mir-279e∼2944c* (Fig. 5B, black arrowheads). These clusters encode a combination of conserved and *Tribolium-*specific microRNAs (Fig. 5C); clusters encoding homologs of the conserved mir-9, mir-2944, mir-279, mir-3791, and mir-309 families are found in other insect lineages and represent one of the most extreme examples of microRNA gain, loss, duplication, and rearrangement reported to date (Ninova et al. 2014). Previous studies by us and others have shown that members of one of these clusters (*mir-309∼6*) in *Drosophila* are strongly up-regulated in the early fly blastoderm (Biemar et al. 2005; Ninova et al. 2014), and that members of the homologous clusters in *A. mellifera* (*mir-3478∼318*) and mosquitoes (*mir-309∼286*) are also highly expressed in early embryos (Zondag et al. 2012; Hu et al. 2014). In addition, the mosquito-specific *mir-2941∼2946* cluster, whose 3′ mature sequences have the same seed as miR-3889-3p in *T. castaneum*, are the most abundant microRNAs in the early stages of development (Hu et al. 2014). We detect nascent primary transcripts of *mir-9d∼3791* and *mir-3889∼3843* in the early blastoderm nuclei of *T. castaneum* by in situ hybridization (Supplemental Fig. 4A). Taken together, these data suggest that, despite multiple rearrangements, the early zygotic onset of expression of these groups of microRNAs is conserved. Interestingly, we detect nascent *mir-9d∼3791* transcripts much later in development, in serosal nuclei, suggesting a novel role of members of this cluster (Supplemental Fig. 4B).

While the levels of *Tribolium-*specific microRNAs are low during most developmental stages, the majority display a coordinated sharp increase in expression at the undifferentiated blastoderm stage (8–16 h) (Fig. 5B,D). Without exception, the blastoderm-specific pool of nonconserved microRNAs belong to the multicopy novel clusters encoding divergent members of the mir-3851, mir-3836, and other *Tribolium-*specific microRNA families described above (Fig. 5B, gray arrowheads; also see Fig. 1). The expression patterns of these microRNA clusters suggest that they are up-regulated for a discrete period of time, immediately following the onset of zygotic transcription in the blastoderm, and are rapidly extinguished shortly afterward. To test this, we assessed the expression of putative nascent transcripts corresponding to selected highly expressed nonconserved microRNA clusters by in situ hybridization (Fig. 5E). Due to the high sequence similarity between some regions, we expect that a subset of probes would cross-hybridize and detect transcription from more than one locus, and indeed this is the case for the mir-3851a-1 region (Fig. 5E, third panel). The data show that the multicopy microRNA clusters are ubiquitously expressed in all blastoderm nuclei, but surprisingly, their expression is limited to a very narrow time interval from the 8th–9th to the 11th cleavage division.

### Abundant early expressed microRNAs target maternally loaded and zygotically down-regulated genes

Very early expressed microRNAs have been shown to play a role in the clearance of maternally deposited transcripts in both *Drosophila* and zebrafish (Giraldez et al. 2006; Bushati et al. 2008). However, the microRNAs involved in this process in the two species (*mir-309∼6* and *mir-430* clusters, respectively), are not homologous, and microRNA-dependent maternal transcript clearance is thought to be a convergent phenomenon. Nonetheless, a common feature is that in both taxa these microRNAs are encoded in large clusters that have undergone multiple duplications and diversification. The most highly expressed microRNAs in the early embryo of *T. castaneum* are also encoded in large clusters, including *mir-9d∼3791* (homologous to the fruit fly *mir-309∼6* cluster [Ninova et al. 2014]), *mir-279e∼2944c*, and *mir-3889∼3843* (Fig. 5B,C), as well as other large species-specific clusters discussed above (Figs. 1, 5B). Thus, we asked whether these or other *T. castaneum* microRNAs might be involved in maternal transcript clearance.

**Figure 6.**
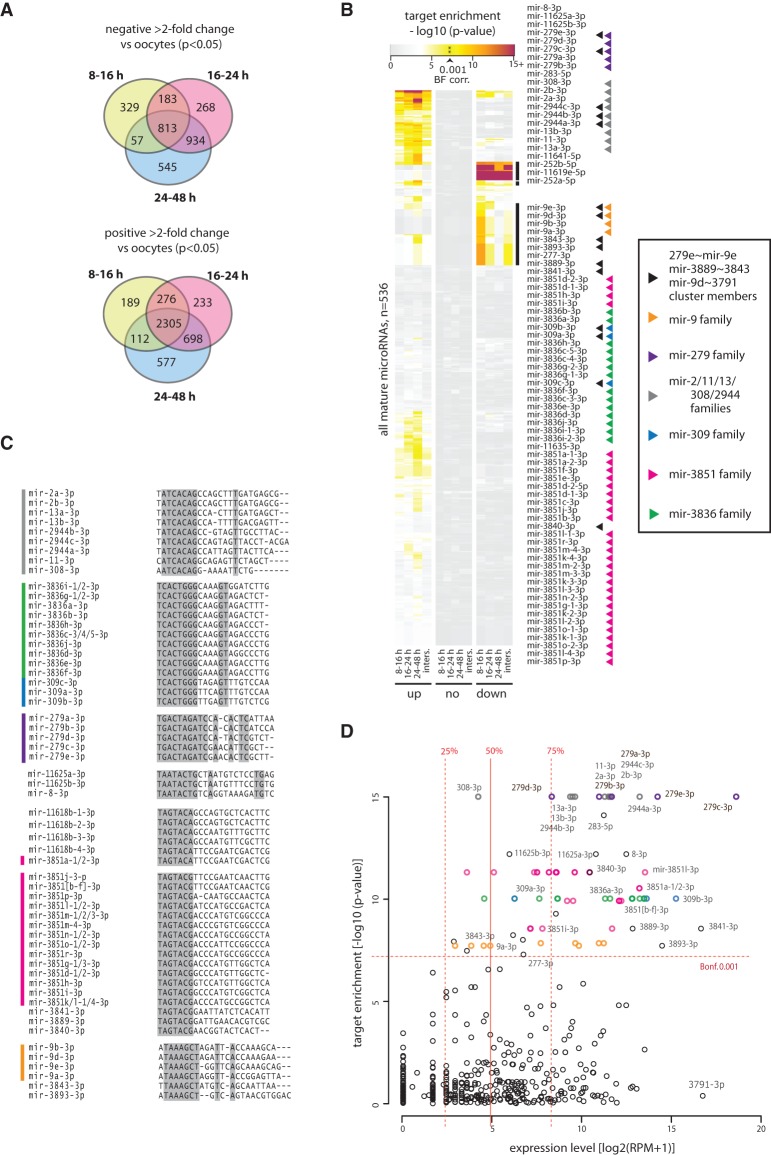
MicroRNA targeting of maternally deposited transcripts in *T. castaneum* oocytes and developing embryos. (*A*) Venn diagrams showing the number and overlap between greater than twofold up- and down-regulated transcripts (*P* < 0.05) between *T. castaneum* oocytes and the three embryonic time intervals (8–16, 16–24, and 24–48 h). (*B*) Hypergeometric *P*-value for mature microRNA targets enrichment predicted based on canonical site interactions among genes down-regulated (“down”), up-regulated (“up”), and not regulated (“none”) from oocytes to 8–16, 16–24, and 24–48 h embryos. “Inters” denotes the intersection of the three individual sets. The two groups of microRNAs with enrichment *P*-value < 0.001 (Bonferroni corrected) in the down-regulated transcripts are expanded and labeled (*right*). Arrowheads indicate microRNA family and clustering. The values underlying the heat map representation and full data are available as Supplemental Table 3. (*C*) Alignments of mature sequences of major seed families with highly enriched targets in the zygotically down-regulated gene set. Side bars indicate microRNA families color-coded as in *B*. Shading indicates 100% base identity at a given position. MicroRNAs with identical sequences are collapsed on a single line. (*D*) Relationship of microRNA expression and target enrichment in the down-regulated gene set in the 8–16 h embryo. Vertical lines show the median, upper, and lower quantile values, and horizontal line shows the 0.001 *P*-value after Bonferroni correction threshold. Points corresponding to the microRNAs outlined in *B* and *C* are color-coded accordingly. Selected microRNAs are labeled.

To determine the maternally deposited mRNA complement, and its fate after zygotic genome activation, we used RNA sequencing to estimate protein-coding transcript levels in unfertilized oocytes, early blastoderm embryos (8–16 h), embryos at the stage of blastoderm differentiation, gastrulation, and serosal closure (16–24 h), and embryos at the stage of germband elongation and segmentation (24–48 h). The resulting ∼300 million paired-end reads were mapped against the *T. castaneum* genome and transcriptome, detecting at least one fragment for 15,221 out of the 16,503 annotated protein-coding genes (92%). Gene expression levels were highly similar between replicates (*r* > 0.96), and differential expression analyses show that a large number of transcripts significantly change their abundance between different intervals (Fig. 6A; Supplemental Fig. 5). In particular, we find a large number of transcripts that are highly up-regulated between embryonic development and oocytes, likely reflecting developmental processes activated in the early embryo. A substantial number of transcripts also display a smaller yet significant negative change in their levels with the progression of embryogenesis, indicating maternal transcript clearance. We predicted the putative microRNA targets of the *T. castaneum* transcripts by two different microRNA target prediction approaches—detection of canonical microRNA-target binding sites (Bartel 2009), and using the miRanda algorithm, which takes into account sequence complementarity and RNA-RNA duplex free energy (Enright et al. 2003). These methods resulted in 12,565 and 12,660 predicted microRNA targets among the 13,412 genes with available 3′ UTR annotations, respectively, with 144,579 individual microRNA-target pairs overlapping between the two sets, and 276,412 and 241,723 pairs unique for each set. We then calculated whether the targets of individual mature microRNAs are enriched among the protein-coding transcripts that are significantly (more than twofold) down-, up-, or not regulated between oocytes and embryos. Despite the poor overlap of individual target sites between the target prediction methods, the overall trends in microRNA target enrichment are highly consistent. Distributions of the hypergeometric *P*-values of target enrichment based on canonical interactions are shown in Figure 6B, and the complete data sets for both target prediction algorithms, as well as analyses performed using an alternative differential expression algorithm, are available as Supplemental Table 3. The significance of microRNA target site enrichment within zygotically up- and down-regulated genes was additionally assessed by permutation tests, yielding similar results (Supplemental Table 3). The data show that genes that are down-regulated between oocytes and embryos, particularly in the early blastoderm (8–16 h), are strongly enriched in targets of a specific set of microRNAs. In general, these microRNAs do not target a significant proportion of genes that are up-regulated or that maintain their expression. We further assessed the expression levels, sequence, genomic localization, and evolutionary relationships of the microRNAs that specifically target zygotically down-regulated genes but not up-regulated genes (enrichment of targets in the down-regulated set with *P* < 0.001 after Bonferroni correction). First, we note that this set of microRNAs consists of multiple paralogs from a small number of families, inferred by manual inspection of hairpin multiple sequence alignments (Fig. 6B labels; Supplemental Fig. 2). Altogether, this set of microRNAs includes 15 of the 16 members of the *mir-279e∼2944c*, *mir-3889∼3843*, and *mir-9d∼3791* clusters, as well as novel and previously annotated members of the mir-3851 and mir-3836 families, which are uniquely up-regulated upon the activation of zygotic transcription. Sequence comparisons further showed that many microRNAs that lack overall sequence similarity have identical 6-,7-, or 8-mer seed regions (herein referred to as “seed families”) (Fig. 6C), and thus have highly similar predicted canonical target sets. Major seed families include AGUACG (3p arms of mir-3851b-q, mir-3889, mir-3840, and mir-3841), AGUACA (3p arm of mir-3851a and mir-11618b), CACUGG (3p arms of mir-3836 and mir-309/3 families), AUCACA (3p arms of mir-2944, mir-2/13, mir-11, and mir-308; complementary to the K-box motif) (Lai et al. 1998), UAAAGC (3p arms of mir-9, mir-3893, and mir-3843; complementary to the Brd-box motif) (Leviten et al. 1997); GACUAG (3p arms of miR-279a to e), and AAUACU (3p arms of mir-8 and novel microRNAs mir-11625a/b). In addition, zygotically down-regulated genes are also enriched in target sites of miR-283-3p, miR-252-3p, and miR-277-3p. Sylamer analysis (van Dongen et al. 2008) showed that of all possible 6-mer nucleotide words, motifs complementary to the seed regions of microRNAs from the above families are among the most highly and significantly enriched motifs in the 3′ UTR of the zygotically down-regulated genes (Supplemental Table 4).

Assessment of microRNA expression levels in the context of their targeting properties showed that the vast majority of microRNAs targeting down-regulated genes in the blastoderm are among the most highly expressed microRNAs at that stage (Fig. 6D). The reciprocity between specific microRNA up-regulation and the down-regulation of their targets in the early blastoderm strongly suggests that these microRNAs are involved in maternal transcript clearance in *T. castaneum*. We also note that a different set of highly expressed and conserved microRNAs, including miR-9-5p, mir-263, and mir-276, target a substantial fraction of genes up-regulated during embryogenesis, reflecting a likely role for these microRNAs in other developmental processes (Supplemental Table 3).

Previous findings in *D. melanogaster* showed that highly expressed members of the *mir-309∼6* cluster in the early embryo, including mir-9/4/79, mir-5/6/2944, mir-3/309, and mir-279/286, are involved in maternal transcript turnover (Bushati et al. 2008). Our results suggest that these microRNA families have a conserved role in maternally deposited transcript regulation in the early embryo of holometabolous insects. Furthermore, we identify several additional, *Tribolium*-specific microRNA families involved in the process, including mir-3889, mir-3840, mir-3841, and mir-3851 families. We therefore suggest that the microRNA repertoire involved in maternal transcript clearance has diverged.

## Discussion

### Abundance and modifications of maternally deposited microRNAs

Oocyte maturation is accompanied by the deposition of a large number of protein and RNA factors, including small RNAs from the microRNA and piRNA classes. In *Drosophila*, maternal piRNA deposition is required for the inheritance of transposon defense (Brennecke et al. 2008). The role of maternally provided microRNAs, on the other hand, is not well understood: microRNAs can be detected in *Drosophila* oocytes, but it is unclear whether these are products generated at earlier stages of gonadal development, or if their deposition is required for subsequent events in embryonic development. For instance, genetic knockout of the maternally provided *mir-310∼mir-313* cluster in *Drosophila* does not result in developmental defects (Tsurudome et al. 2010; Pancratov et al. 2013). MicroRNAs are not present at high levels in small RNA libraries of zebrafish, *Xenopus*, and mouse oocytes, and the roles of maternally deposited microRNAs in these species are not well understood (Chen et al. 2005; Watanabe et al. 2005; Ohnishi et al. 2010; Lee et al. 2014).

As in vertebrates, the cloning frequency of microRNAs in oocytes of *Tribolium* is very low compared to subsequent developmental stages. However, absolute quantification of *T. castaneum* maternally deposited microRNAs shows a concentration of ∼0.2 fmol per oocyte, which is commensurate with previous estimates of microRNA copy numbers in mammalian cells (Bissels et al. 2009) and likely reflects physiologically relevant levels. Thus, the underrepresentation of microRNAs in the *T. castaneum* oocyte libraries is not due to absence of maternal loading but to very high levels of piRNAs. RNA sequencing shows high levels of transposon activity in the *Tribolium* embryo, and we speculate that the high abundance of piRNAs is related to this observation (M Ninova, S Griffiths-Jones, M Ronshaugen, in prep.).

The metabolism of maternally provided microRNAs is not well understood. It was recently demonstrated that a substantial proportion of the microRNA complement in oocytes is 3′-end adenylated post-transcriptionally. Since this modification enhances microRNA degradation, it provides a plausible mechanism to control clearance of maternally loaded microRNAs (Lee et al. 2014). Consistent with this possibility, we detect high levels of 3′-end oligoadenylated microRNAs in *Tribolium* and *Drosophila* oocytes. Interestingly, only specific mature microRNAs are modified at high levels, but this is not obviously determined by microRNA sequence (Lee et al. 2014). Our data demonstrate that the specificity of microRNA adenylation in oocytes is similar for abundant microRNA orthologs between *Drosophila* and *Triboliium*, suggesting that the mechanism that regulates this process is conserved among holometabolous insects, across at least ∼300 million years of evolution. Further work is required to elucidate the molecular basis and developmental effects of this process.

### MicroRNA-mediated maternal transcript clearance in *T. castaneum*

One of the earliest events during animal development is the degradation of maternally deposited transcripts in the egg and the activation of the zygotic genome—a process termed the maternal-to-zygotic transition (MZT) (for review, see Tadros and Lipshitz 2009). MicroRNAs have been implicated in the MZT of both invertebrates and vertebrates. However, the data suggest that involvement of microRNAs in this function has evolved convergently in the two clades (Bushati et al. 2008). In zebrafish, among the first zygotically expressed transcripts is a large cluster encoding over 50 mir-430 paralogs, which target a significant fraction of the maternally provided mRNAs (Giraldez et al. 2006). In *Drosophila*, a similar role was proposed for the early expressed *mir-309∼6* cluster (Bushati et al. 2008). Genetic knockout of the *mir-309∼6* cluster results in the delayed degradation of a number of maternally provided mRNAs but does not cause significant embryonic defects (Bushati et al. 2008). We speculate that members of the *mir-309∼6* cluster are not the only microRNAs involved in maternal transcript regulation in *Drosophila*, as other microRNAs from the same seed families—including miR-2/11/13-3p, miR-279/996-3p, and mir-9 paralogs—are present in the early embryo.

We have identified a number of conserved and species-specific microRNAs in *T. castaneum* that are highly expressed in the early embryo and target a significant fraction of down-regulated maternal transcripts during the MZT. Thus, the data suggest that microRNAs are involved in the degradation of maternally deposited transcripts in this lineage. The conserved microRNAs involved in maternal transcript down-regulation in the blastoderm of the flour beetle include homologs of the *Drosophila mir-309∼6* cluster members and other microRNAs with identical seeds. Taken together, the data suggest that microRNA-mediated maternal transcript degradation by the 3p mature arms of microRNAs from the seed families AUCACA (mir-2944/5/6, mir-2/13 and mir-11), UAAAGC (mir-9/79/4 family), and CACUGG (mir-309/3 family) is a conserved feature in holometabolous insects. mir-279 family members are very highly expressed in the early embryo and show one of the strongest target enrichment values among the down-regulated transcripts in the early embryo. In addition, we observed high expression levels and target site enrichment in the zygotically down-regulated genes targeted by mir-8 and mir-283, and to a lesser extent, mir-277 and mir-252. The roles of these microRNAs in maternal transcript clearance in *Drosophila* have not been previously addressed; further studies are required to determine whether the involvement of these deeply conserved microRNAs in the MZT is conserved in insects or represents a *Tribolium-*specific co-option.

Several *T. castaneum-*specific microRNAs also target a significant fraction of the maternally deposited transcripts that decrease at the blastoderm stage. These include the multicopy microRNA families mir-3836 and mir-3851 and four hairpins encoded in the *mir-3889∼3843* and *mir-9d∼3791* clusters: mir-3889, mir-3840, and mir-3841, and mir-3893. Notably, 3p products corresponding to the seed families of mir-3851a (AGUACA) and mir-3851b to q, mir-3889, mir-3840, and mir-3841 (AGUACG) are not found in *Drosophila*, suggesting that these microRNA-target interactions are a diverged feature between these insect lineages.

Taken together with previous studies in *Drosophila*, findings in *T. castaneum* suggest that microRNA-mediated maternal transcript degradation is a conserved mechanism in holometabolous insects, but the precise microRNAs participating in this process differ somewhat between species. MicroRNAs involved in maternal transcript clearance in vertebrates (Giraldez et al. 2006; Lund et al. 2009) have no sequence similarity or common seed motifs with any of the insect microRNAs, illustrating the likelihood of convergence in this process (see below). Nevertheless, comparisons of the microRNAs in the MZT in different organisms reveal the common phenomenon of large, fast-evolving microRNA polycistrons involved in this process.

### Dynamic evolution of early expressed microRNAs

Our analysis of the genomic positions, sequences, and targeting properties of the early expressed *T. castaneum-*specific microRNAs reveal complex relationships. The mir-3851 and mir-3836 families, and novel mir-8 seed family members, are found in large and diverse clusters located in multiple genomic positions. These microRNAs are not colocalized with any conserved microRNAs, but other members from these seed families, such as mir-309, have deeper evolutionary origins and are clustered with other conserved microRNAs. MicroRNA hairpins are short, and thus any putative fast-evolving sequences can diverge to the point at which they cannot be confidently identified as homologs. On the other hand, the formation of microRNAs with identical seeds (microRNA convergence) may be common, as the microRNA seed region is very short, and new hairpins often emerge de novo in animal genomes. Despite their high degree of divergence in terms of encoded hairpin copy number, family, and sequence, the *T. castaneum-*specific microRNA clusters display a very similar temporal expression pattern spanning only a few rounds of cell division after the initiation zygotic transcription. We propose that newly emerged microRNAs with convergent seeds and similar expression patterns to existing microRNAs are more likely to be retained, as they “mimic” the existing microRNA and thus do not cause significant transcriptome perturbations by down-regulating new transcripts. In the light of this hypothesis, one explanation for the origin of the mir-3836 and mir-3851 clusters is that their founding members emerged from random hairpins in early activated regions and subsequently duplicated and diversified. Alternatively, if microRNAs with identical seeds are considered to be highly diverged paralogs, we can speculate that the mir-3836 and mir-3851-encoding clusters, and the three clusters encoding members of the conserved mir-5/6/2944, mir-9/4, mir-279/286, and mir-309/3 families, have common origins but have significantly diverged via multiple duplications, rearrangements, and losses (including the acquisition of a mir-8 paralog that rapidly diverged). These scenarios of cluster evolution are not mutually exclusive: It is likely that some seed families are evolutionarily related, while others emerged by convergence. Either way, the evolutionary patterns of the early expressed microRNAs are uniquely dynamic.

Our previous work demonstrated that one characteristic of the early *Drosophila* embryo is high levels of fast-evolving microRNAs (Ninova et al. 2014). Data from *T. castaneum* now suggest that the early embryonic expression of fast-evolving and evolutionarily younger microRNAs is not restricted to *Drosophila* but represents a conserved feature of holometabolous insects. Studies in other organisms have also suggested that early embryogenesis is permissive or robust to evolutionary change in the transcriptome compared to later stages of development: In *Drosophila,* vertebrates, and plants, the early embryonic transcriptome is, on average, younger, faster evolving, and characterized with higher variation in orthologous gene expression (Domazet-Lošo and Tautz 2010; Kalinka et al. 2010; Quint et al. 2012; Heyn et al. 2014). Even though the underlying causes of this phenomenon are elusive, our results suggest that the apparent flexibility of the molecular networks active in early development also impacts the evolution of the microRNA complement expressed at that stage.

## Methods

### Animal husbandry, sample collection, and deep sequencing

*T. castaneum* wild-type adults (Michael Akam, University of Cambridge) were reared following a standard protocol (The Beetle Book: http://wwwuser.gwdg.de/~gbucher1/tribolium-castaneum-beetle-book1.pdf) at 25°C, and wild-type *D. melanogaster* and *D. virilis* were maintained under standard conditions. Details on embryo collection timing are provided in the Supplemental Text. RNA was extracted using a standard TRIzol protocol. Small RNA and RNA libraries were constructed using the Illumina TruSeq Small RNA Sample Prep and TruSeq Stranded mRNA Sample Prep kit, respectively. Libraries were assessed using the Agilent 2200 TapeStation and sequenced on the Illumina MiSeq (embryonic small RNA sequencing) or the Illumina HiSeq 2000 (oocytes small RNA sequencing and whole-transcriptome sequencing) platforms in the University of Manchester Genomic Technologies facility.

### Embryo fixation, immunohistochemistry, and in situ hybridization

Embryos were dechorionated, fixed, and devitellinated using a standard protocol. Whole-mount fluorescent in situ hybridization with ∼1 kb-long DIG-labeled antisense RNA probes and antibody staining procedures were performed according to the protocol in Kosman et al. (2004), but omitting the proteinase K treatment step. Primers for RNA probe synthesis templates and antibodies used for detection are listed in Supplemental Table 5. Images were visualized by confocal microscopy on an Olympus FV1000, and image stacks were processed with Fiji (Schindelin et al. 2012).

### Small RNA sequencing data analysis and microRNA prediction

Adapter sequences were trimmed from the *T. castaneum* small RNA reads using the Cutadapt tool (http://code.google.com/p/cutadapt/), retaining reads longer than 16 nt. Reads were first filtered against *T. castaneum* tRNA genes predicted using tRNAscan-SE (v1.3) (Lowe and Eddy 1997), and then mapped to the latest version of the *T. castaneum* genome assembly (r4.0) using Bowtie (v1.0) (Langmead et al. 2009) with the following parameters: -v 1 –a --best –strata –m 5. Mapped reads were used as input to two independent microRNA discovery methods—mirdeep2 (Friedländer et al. 2008) and an implementation of the method described in Marco et al. (2010). Newly discovered microRNAs were submitted to miRBase (Kozomara and Griffiths-Jones 2014). MicroRNA read counts were calculated, correcting for mapping to multiple locations, and expression was normalized as reads per million mapped to the genome. *D. melanogaster* and *D. virilis* oocyte small RNA libraries were analyzed as previously (Ninova et al. 2014).

For 3′ nontemplate end additions, reads that did not map to the genome with 0 mismatches were sequentially trimmed by 1 nt from their 3′ end and remapped. Upon each iteration, perfectly mapping reads were retained. Resulting trimmed sequences corresponding to full-length microRNAs were analyzed using a custom Perl script (see Supplemental Material).

### MicroRNA evolutionary conservation

*T. castaneum* microRNAs were grouped into families based on best BLASTN hits (-word_size = 4) (Altschul et al. 1990) and manual inspection and editing of the resulting alignments using RALEE (Griffiths-Jones 2005). Curated alignments were used to build covariance models, and these models were searched against the genomes of *Dendroctonus ponderosae*, *D. melanogaster*, *D. virilis*, *A. mellifera*, and *Bombyx mori* using INFERNAL (Nawrocki et al. 2009) with an *E*-value cutoff of 1. MicroRNAs with no hits were considered species-specific. Hits scoring below this threshold were added to previous alignments and manually inspected for hairpin folding and homology.

### RNA sequencing data analysis and differential expression

Paired-end transcriptome data were mapped to the *T. castaneum* genome (r4.0) using TopHat (Trapnell et al. 2009) with default parameters and supplying the currently available protein-coding gene annotations (iBeetle, http://bioinf.uni-greifswald.de/tcas/genes/annotation/). Gene expression counts were obtained using htseq-count (Anders et al. 2015), and differential gene expression between oocytes, 8–16-, 16–24-, and 24–48-h embryos was assessed using the DESeq R package (Anders and Huber 2010). Gene expression changes with *P*-values smaller than 0.05 after Benjamini–Hochberg correction were considered as significant. We further validated these results using the Cuffdiff program from the Cufflinks package (Trapnell et al. 2010) as an alternative approach to estimate gene differential expression (Supplemental Table 3).

### MicroRNA target predictions and enrichment analyses

MicroRNA target sites in the annotated 3′ UTR of *T. castaneum* protein-coding genes (r4.0, iBeetle Database, http://ibeetle-base.uni-goettingen.de/) were predicted using two independent target prediction algorithms with the default parameters—an implementation of the canonical site target pairing as described in Bartel (2009), provided by Antonio Marco, and miRanda (Enright et al. 2003). When multiple transcripts per gene were present (7% of all annotations), different UTRs were merged; we note that considering individual transcripts separately produces very similar results (data not shown). Target enrichment was independently assessed by hypergeometric (phyper R function) and permutation tests. For the latter, random samples of equal sizes to the significantly up- and down-regulated gene sets between oocytes and later stages were drawn without replacement 1000 times, and numbers of microRNA targets were calculated. *P*-values were corrected for multiple testing using the Bonferroni correction. Sequence motif enrichment in transcript UTRs was assessed by sylamer (van Dongen et al. 2008).

## Data access

All RNA sequencing data from this study have been submitted to the NCBI Gene Expression Omnibus (GEO; http://www.ncbi.nlm.nih.gov/geo/) under accession number GSE63770.

## Supplementary Material

Supplemental Material
