# Supplementary material for: MicroRNA evolution, expression, and function during short germband development in Tribolium castaneum
Source: Genome Res. 2016 Jan;26(1):85–96. doi: 10.1101/gr.193367.115 (PMC4691753; doi:10.1101/gr.193367.115)
Supplement: Supplemental Material [file supp_26_1_85__index.html]

MicroRNA evolution, expression and function during short germband development in Tribolium castaneum — MicroRNA evolution, expression, and function during short germband development in Tribolium castaneum — MicroRNA evolution, expression, and function during short germband development in Tribolium castaneum — Supplemental Material 

# MicroRNA evolution, expression, and function during short germband development in *Tribolium castaneum*

## Supplemental Material

**Files in this Data Supplement:**

- Supplemental Figures.pdf
- Supplemental scripts.zip
- Supplemental Table1.xlsx
- Supplemental Table2.xlsx
- Supplemental Table3.xlsx
- Supplemental Table4.xlsx
- Supplemental Table5.xlsx
- Supplemental Text.docx
