# Supplementary material for: MicroRNA evolution, expression, and function during short germband development in Tribolium castaneum
Source: Genome Res. 2016 Jan;26(1):85–96. doi: 10.1101/gr.193367.115 (PMC4691753; doi:10.1101/gr.193367.115)

Supplemental Figure 1

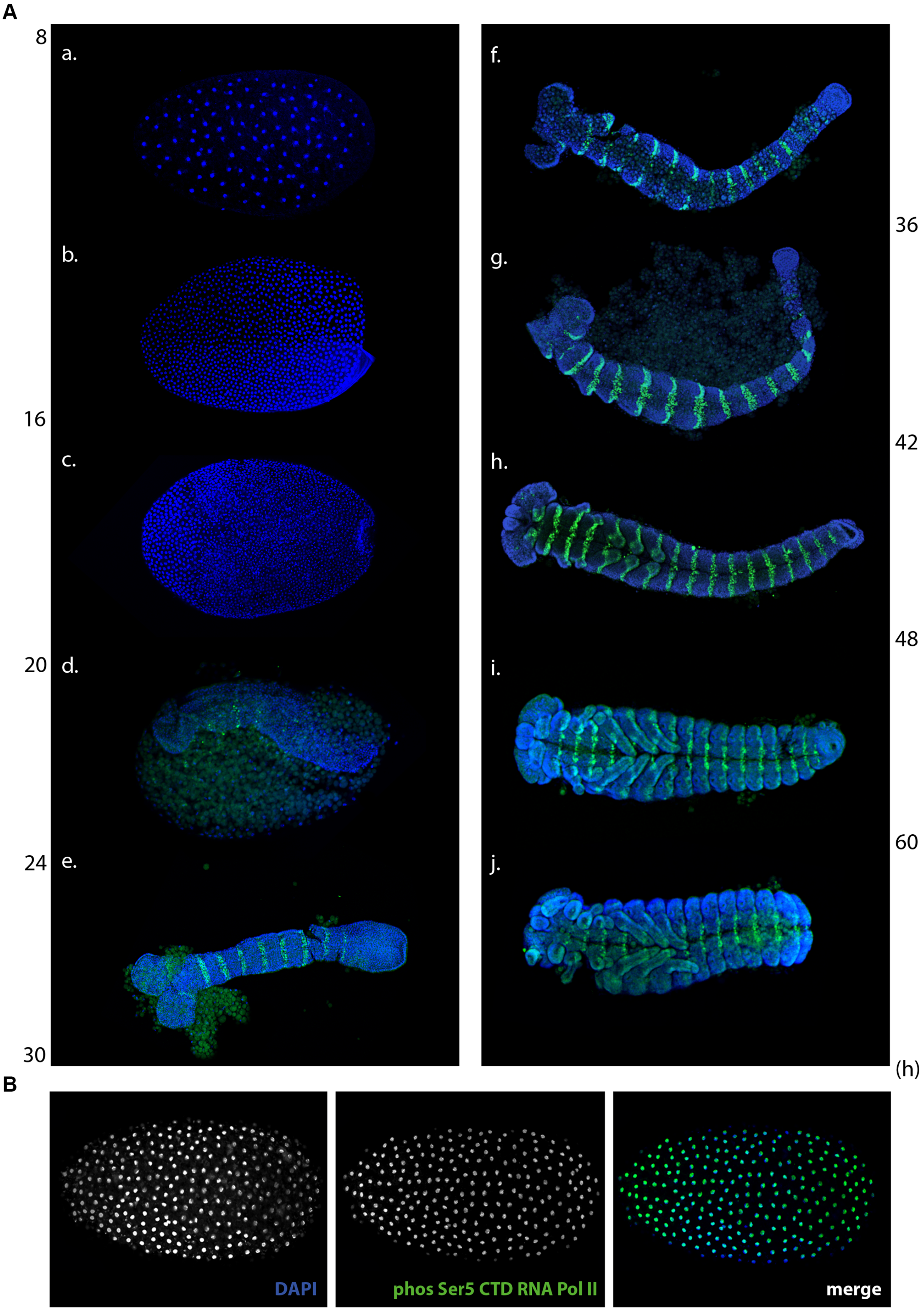

Supplemental Figure 2

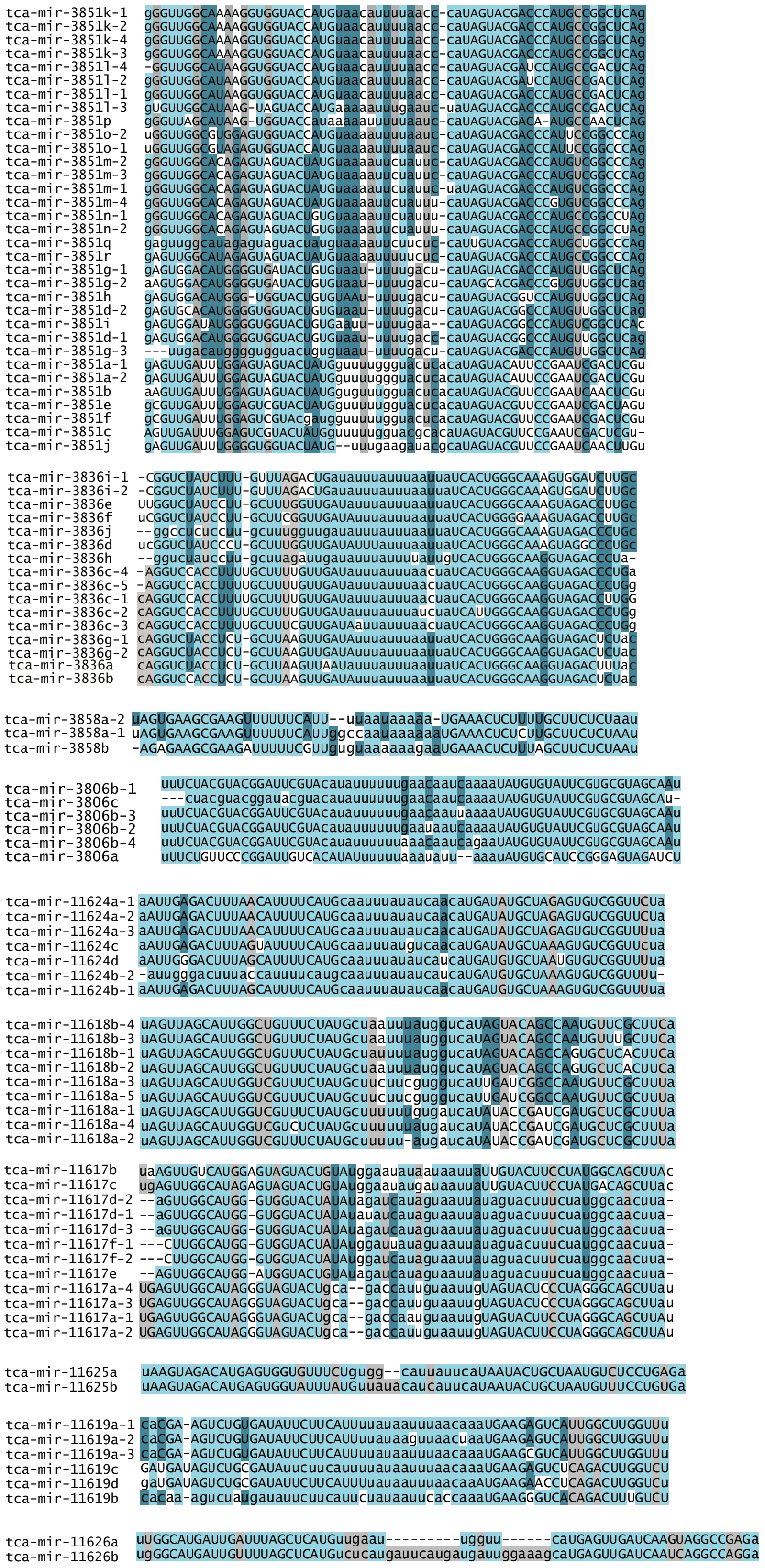

Supplemental Figure 3

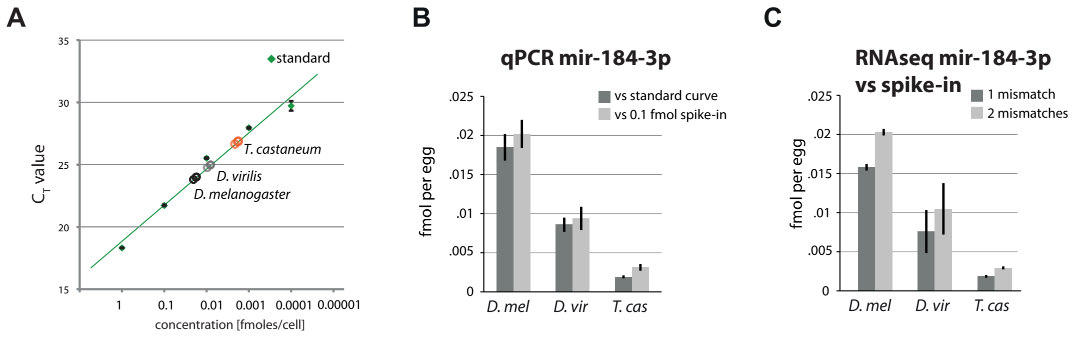

Supplemental Figure 4

A

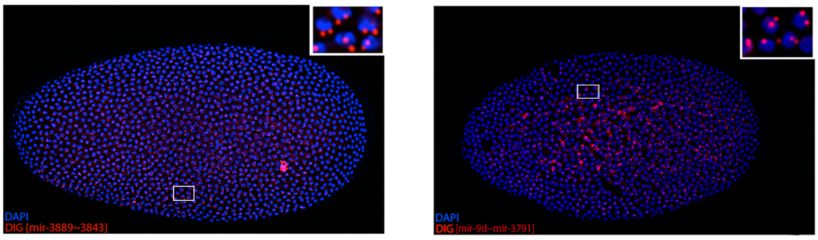

B

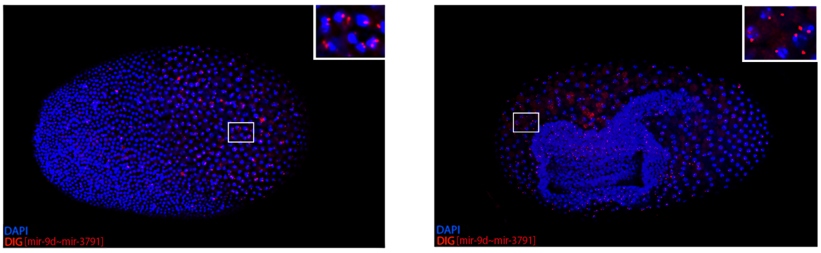

Supplemental Figure 5

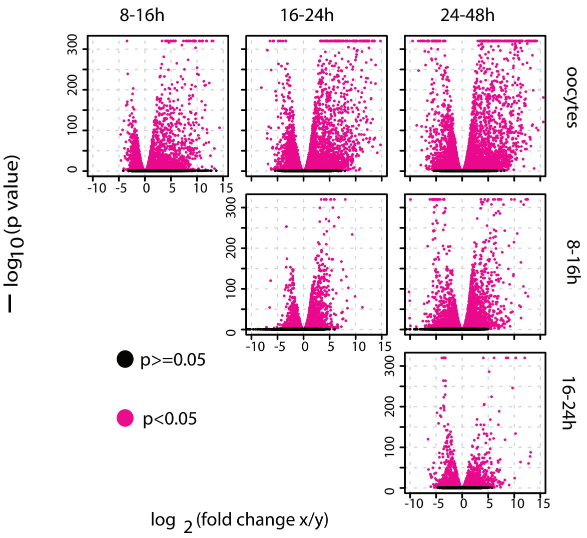

Supplement: Supplemental Material [file supp_gr.193367.115_Supplemental_Figures.pdf]
