# Supplementary material for: MicroRNA evolution, expression, and function during short germband development in Tribolium castaneum
Source: Genome Res. 2016 Jan;26(1):85–96. doi: 10.1101/gr.193367.115 (PMC4691753; doi:10.1101/gr.193367.115)
Supplement: Supplemental Material [file supp_gr.193367.115_Supplemental_Text.docx]

## Supplemental Text

### Timing of events during *T. castaneum* development

Establishing the precise timing of events during *T. castaneum* embryogenesis is an important prerequisite for the preparation of small RNA sequencing libraries representing discrete developmental stages. Previous studies provided developmental event tables for early stages of *T. castaneum* at 30 and 32°C (Handel et al. 2000; Benton et al. 2013). However, as development at these temperatures is relatively fast while female fertility is relatively low, obtaining sufficient numbers of precisely staged embryos under these conditions is technically challenging. To overcome this, we maintained *T. castaneum* adults and embryos at 25°C, which prolonged embryonic development and allowed us to obtain a larger number of embryos of narrower time intervals. To establish the timing of developmental events at this temperature, we collected embryonic samples from different overlapping 6 or 8 hour time intervals after oviposition, and staged them using morphology and the expression of the protein product of *engrailed*, which marks the progression of segmentation. Representative images of selected time intervals are shown in Supplemental Figure 1A. We can clearly identify 2-cell embryos, and we find that the stages of embryos in each sample were closely related, ruling out egg retention by females. *T. castaneum* embryogenesis begins with nuclear divisions without cell divisions; approximately 8 hours after fertilization nuclei migrate to the periphery. In the periphery, nuclei continue to divide uniformly for an additional 8 hours, during which cellularization occurs (Handel et al. 2000). After the 16 h time point, cells from the so far uniform blastoderm start to segregate forming the prospective embryonic tissues and serosa. This segregation, together with the formation of the primitive pit, occur between 16-20 h at 25ºC. Gastrulation and mesoderm formation are also thought to begin at that time (Handel et al. 2005). The next ~4 hour interval (20-24 h) covers embryonic rudiment folding and the serosal window closure, and first stripes of *engrailed* emerge at this stage. The germband then starts to markedly grow from the posterior of the embryo, accompanied by the appearance of additional stripes of *engrailed*. 36-42 h after fertilization germband elongation is completed, and the development of appendages begins. Appendages and the pre-gnathal regions of the embryo continue to develop as the germ band thickens and shortens. At about 60-66h hours post fertilization, cuticle secretion begins, and larvae hatch approximately 6 days after fertilization.

A delay between fertilization and zygotic gene expression is common in many animals, with the protein and RNA component of the transcriptionally silent early embryo being maternally deposited in the egg. To establish the precise timing of zygotic transcription activation in *Tribolium,* we immunostained early embryos with an antibody that detects a phosphorylated residue only in active RNA polymerase II (Supplemental Figure 1B). We detect ubiquitous active transcription after the 8^th^ cleavage division, when nuclei migrate from the interior to the periphery of the embryo.

## Supplemental Methods

**Embryonic sample collection for RNA sequencing**

Unfertilized eggs were obtained from virgin females isolated at the pupal stage that were allowed to lay for 4 hours. RNA was extracted from fixed number (30) of manually-picked eggs. The final RNA pellet was resuspended in equal volumes of cocktail containing and synthetic homologs of *C. elegans* miR-35-3p, miR-56-3p and miR-230-3p, at 0.1, 0.01 and 0.001 femtomoles per egg final concentrations.

For *T. castaneum* small RNA developmental profiles, we collected embryonic samples representing 0-5 h (in two replicates), 8-16 h, 16-20 h, 20-24 h, 24-34 h, 34-48 h and 2-6 d time intervals at 25°C in two biological replicates. For whole transcriptome RNA sequencing, we used two biological replicates of *T. castaneum* unfertilized eggs, 8-16 h, 16-24 h and 24-48 h embryos. Accurate staging was verified by microscopic inspection of an aliquot of each sample.

### Absolute quantitation of microRNA expression in oocytes

We determined the absolute abundance of miR-184-3p per cell in *Drosophila* and *Tribolium* oocytes, and used these values as endogenous references of microRNA expression in small RNA sequencing libraries. This microRNA was chosen for its relative abundance and invariant sequence between the three species, thus avoiding potential sequence-related biases in quantitation. First, we measured the absolute amount of miR-184-3p per cell by qPCR by standard curve of 10-fold dilutions of synthetic 5’ phosphorylated miR-184-3p oligonucleotides. To do this, we extracted RNA from 10 oocytes of each species, and used 1/10 of the extracted RNA as an input for cDNA synthesis. Results were highly reproducible among the three replicates per species (Supplemental Figure 3A), and similar values were obtained upon normalizing the miR-184-3p concentration with respect to the C_T_ values of the 0.1 fmoles/egg spike-in (Supplemental Figure 3B). To assess whether these quantitative relationships are valid in deep sequencing data, we calculated the ratio of miR-184-3p and the 0.1 fmoles/egg spike-in (cel-miR-35-3p) read counts (Supplemental Figure 3C). Despite small variation depending on the number of mismatches allowed in read mapping to the genome, overall results from qPCR and deep sequencing were highly similar, and both miR-184-3p and the synthetic 0.1 spike-in were used in the downstream analysis as an endogenous and exogenous reference, respectively. The lower concentration spike-ins had very few reads in the deep sequencing experiments and were therefore ignored for further analysis.

## Supplemental Figure Legends

**Supplemental Figure 1. Embryonic development of *T. castaneum* at 25ºC.** **A.** Samples were taken at 6 or 8 hour time intervals during the first 66 h of *T. castaneum* development and immunostained for *engrailed*. Images are oriented with the anterior to the right. Representative pictures of each time point or interval (numbers) are shown. a,b) the earliest and the latest stage of the 8-16 h time interval, cleavage divisions and uniform blastoderm; c) 16-20 h, differentiating blastoderm forming embryonic rudiment and serosa; d) 20-24 h, serosal window closure and first stripes of engrailed; e) to f) 24-36 h, germband elongation until full extension; g) and h) 36-48h, fully extended germband, appendages emerging on the gnathal, thoracic and first abdominal segments. i) and j) 48-66h, post-segmentation embryo, appendages continue to develop as the germband shortens. **B.** First interphase at which ubiquitous active (phospho-serine 5 CTD) RNA polymerase II is detected (green). Nuclei are stained with DAPI (blue).

**Supplemental Figure 2. Multiple sequence alignments of the *Tribolium* microRNA families.** Alignments include microRNA precursor sequences, with the two mature arms in upper case. Colors indicate the percentage of nucleotide identity at each position, with light blue corresponding to >80%, dark blue >60% and grey >40% identical nucleotides. Images are generated with RALEE (Griffiths-Jones 2005).

**Supplemental Figure 3. Absolute quantities of miR-184-3p in *T. castaneum, D. melanogaster* and *D. virilis* oocytes. A.** Absolute quantitation of miR-184-3p using standard dilutions of corresponding synthetic oligonucleotides; horizontal axis shows concentrations of RNA standards and vertical axis shows the qPCR (TaqMan) C_T_ values of these standards, and three samples from oocytes of the three species. **B.** Comparisons of miR-184-3p abundance estimates based on standard curve normalization (see A), or relative to the concentration of the 0.1 fmol per cell spiked-in synthetic oligonucleotide. **C.** miR-184-3p abundance relative to the 0.1 fmol per cell spike-in inferred from small RNA sequencing read count ratios calculated allowing one or two mismatches to the reference genomes.

**Supplemental Figure 4. *In situ* hybridization for nascent transcripts of *T. castaneum mir-3889~3843* and *mir-9d~3791* clusters. A.** Blastoderm embryos stained with DIG-labelled (red) antisense RNA probes to *mir-3889~3843* (left) and *mir*-*9d~3791* (right) cluster regions, and DAPI (blue). Nascent transcripts are ubiquitously detectable as nuclear dots. **B**. Embryos at the stage of differentiating blastoderm (left) and serosal closure (right) stained with DIG-labelled (red) antisense RNA probe to the *mir-9d~3791* cluster region and DAPI (blue). Nascent transcripts (nuclear dots) are detectable in serosal nuclei. Example regions are magnified.

**Supplemental Figure 5. Differential gene expression between oocytes and embryonic stages.** Volcano plots show fold change in expression (x-axis) versus differential expression p-value (y-axis) of gene expression (FPKM) in samples from *T. castaneum* oocytes, 8-16 h, 16-24h and 24-48h embryos, estimated by Cuffdiff. Transcripts with a significant fold difference between samples (p<0.05) are coloured.
